# Exposure to Ambient Air Pollution and the Risk of Inflammatory Bowel Disease: A European Nested Case–Control Study

**DOI:** 10.1007/s10620-016-4249-4

**Published:** 2016-07-26

**Authors:** Jorrit L. Opstelten, Rob M. J. Beelen, Max Leenders, Gerard Hoek, Bert Brunekreef, Fiona D. M. van Schaik, Peter D. Siersema, Kirsten T. Eriksen, Ole Raaschou-Nielsen, Anne Tjønneland, Kim Overvad, Marie-Christine Boutron-Ruault, Franck Carbonnel, Kees de Hoogh, Timothy J. Key, Robert Luben, Simon S. M. Chan, Andrew R. Hart, H. Bas Bueno-de-Mesquita, Bas Oldenburg

**Affiliations:** 1 Department of Gastroenterology and Hepatology, University Medical Center Utrecht, P.O. Box 85500, 3508 GA Utrecht, The Netherlands; 2 Institute for Risk Assessment Sciences, Utrecht University, P.O. Box 80178, 3508 TD Utrecht, The Netherlands; 3 Center for Sustainability, Environment and Health, National Institute for Public Health and the Environment (RIVM), P.O. Box 1, 3720 BA Bilthoven, The Netherlands; 4 Department of Gastroenterology and Hepatology, Radboud University Medical Center, P.O. Box 9101, 6500 HB Nijmegen, The Netherlands; 5 Danish Cancer Society Research Center, Strandboulevarden 49, 2100 Copenhagen Ø, Denmark; 6 Department of Environmental Science, Aarhus University, Frederiksborgvej 399, 4000 Roskilde, Denmark; 7 Section for Epidemiology, Department of Public Health, Aarhus University, Bartholins Allé 2, 8000 Aarhus C, Denmark; 8 Paris-Saclay University, Université Paris-Sud, Université de Versailles-Saint-Quentin-en-Yvelines (UVSQ), Centre de Recherche en Épidémiologie et Santé des Populations (CESP), U1018, Institut National de la Santé et de la Recherche Médicale (INSERM), Institut Gustave Roussy, 114 rue Edouard Vaillant, 94800 Villejuif, France; 9 Institut Gustave Roussy, 114 Rue Edouard Vaillant, 94805 Villejuif, France; 10 Department of Gastroenterology, Bicêtre University Hospital, Assistance Publique des Hôpitaux de Paris, 78 Rue du Général Leclerc, 94275 Le Kremlin Bicêtre, France; 11 Environmental Exposure and Health Unit, Department of Epidemiology and Public Health, Swiss Tropical and Public Health Institute, Socinstrasse 57, 4051 Basel, Switzerland; 12 University of Basel, Petersplatz 1, 4001 Basel, Switzerland; 13 MRC-PHE Center for Environment and Health, Department of Epidemiology and Biostatistics, Imperial College London, South Kensington Campus, London, SW7 2AZ UK; 14 Cancer Epidemiology Unit, Nuffield Department of Population Health, University of Oxford, Richard Doll Building, Old Road Campus, Oxford, OX3 7LF UK; 15 Strangeways Research Laboratory, Institute of Public Health, University of Cambridge, Worts Causeway, Cambridge, CB1 8RN UK; 16 Department of Medicine, Faculty of Medicine and Health Sciences, Norwich Medical School, University of East Anglia, Norwich, NR4 7TJ UK; 17 Department of Gastroenterology, Norfolk and Norwich University Hospital NHS Trust, Colney Lane, Norwich, NR4 7UY UK; 18 Department for Determinants of Chronic Diseases, National Institute for Public Health and the Environment (RIVM), P.O. Box 1, 3720 BA Bilthoven, The Netherlands; 19 Department of Epidemiology and Biostatistics, School of Public Health, Imperial College London, South Kensington Campus, London, SW7 2AZ UK; 20 Department of Social and Preventive Medicine, Faculty of Medicine, University of Malaya, Jalan Universiti, 50603 Kuala Lumpur, Malaysia

**Keywords:** Air pollution, Particulate matter, Inflammatory bowel disease, Crohn’s disease, Ulcerative colitis

## Abstract

**Background:**

Industrialization has been linked to the etiology of inflammatory bowel disease (IBD).

**Aim:**

We investigated the association between air pollution exposure and IBD.

**Methods:**

The European Prospective Investigation into Cancer and Nutrition cohort was used to identify cases with Crohn’s disease (CD) (*n* = 38) and ulcerative colitis (UC) (*n* = 104) and controls (*n* = 568) from Denmark, France, the Netherlands, and the UK, matched for center, gender, age, and date of recruitment. Air pollution data were obtained from the European Study of Cohorts for Air Pollution Effects. Residential exposure was assessed with land-use regression models for particulate matter with diameters of <10 μm (PM_10_), <2.5 μm (PM_2.5_), and between 2.5 and 10 μm (PM_coarse_), soot (PM_2.5 absorbance_), nitrogen oxides, and two traffic indicators. Conditional logistic regression analyses were performed to calculate odds ratios (ORs) with 95 % confidence intervals (CIs).

**Results:**

Although air pollution was not significantly associated with CD or UC separately, the associations were mostly similar. Individuals with IBD were less likely to have higher exposure levels of PM_2.5_ and PM_10_, with ORs of 0.24 (95 % CI 0.07–0.81) per 5 μg/m^3^ and 0.25 (95 % CI 0.08–0.78) per 10 μg/m^3^, respectively. There was an inverse but nonsignificant association for PM_coarse_. A higher nearby traffic load was positively associated with IBD [OR 1.60 (95 % CI 1.04–2.46) per 4,000,000 motor vehicles × m per day]. Other air pollutants were positively but not significantly associated with IBD.

**Conclusion:**

Exposure to air pollution was not found to be consistently associated with IBD.

**Electronic supplementary material:**

The online version of this article (doi:10.1007/s10620-016-4249-4) contains supplementary material, which is available to authorized users.

## Introduction

Inflammatory bowel disease (IBD) is a disabling intestinal condition of unclear etiology affecting over two million people in Europe alone [1]. Both Crohn’s disease (CD) and ulcerative colitis (UC), the two main forms of IBD, are thought to result from a complex interplay between the immune system and gut microbiota in genetically susceptible individuals [2]. Yet, the changing epidemiology of IBD supports a key role for environmental factors in the development of this chronic disorder [3].

The incidence of IBD in Western countries has increased over the last century but currently appears relatively stable, whereas a rapid disease emergence is observed in developing nations, where IBD was formerly uncommon [4, 5]. Consequently, industrialization and westernization of lifestyle have been linked to the etiology of CD and UC [3, 6, 7]. Although various risk factors, such as diet [8], have previously been proposed, these do not fully explain the increase in the incidence of IBD and other environmental changes must therefore be involved [9].

Air pollution is associated with industrialization and consists of a complex mixture of different substances, such as particulate matter (PM). Although air pollution has mainly been associated with cardiorespiratory disorders [10], limited experimental and epidemiological data suggest that air pollutants may also exert deleterious gastrointestinal effects [11]. Potential mechanisms by which air pollutants may cause intestinal injury are thought to include direct adverse effects on epithelial cells, alterations in immune responses, or modulation of the gut microbiota [11–14].

Epidemiological studies examining the relationship between air pollution and IBD are scarce. In a case–control study from the UK, children and young adults, but not older individuals, living in areas with high-level exposure to nitrogen dioxide (NO_2_) and sulfur dioxide had an increased risk of developing CD and UC, respectively, indicating possible age-related effects [15]. However, this study was based on regional air pollution estimates, limiting exposure assessment. Therefore, we aimed to investigate the association between residential exposure to ambient air pollution and IBD in a nested case–control study within a multicenter European cohort. Based on previous studies, we hypothesized that air pollution exposure increases the risk of developing CD and UC.

## Materials and Methods

### Study Population

Subjects were identified from the European Prospective Investigation into Cancer and Nutrition (EPIC) study, a large multicenter cohort investigation designed to explore the association between lifestyle and environmental factors and chronic diseases. The methods of the main study were described previously [16]. In the present study, we made use of cases and controls drawn from a subset of this cohort, namely 227,620 initially healthy men and women recruited from five centers in Denmark, France, the Netherlands, and the UK between 1993 and 2000 (Table 1). At baseline, participants provided information on age, gender, habitual diet, and lifestyle factors, including smoking and physical activity, by self-administered questionnaires. Participants’ height and weight were measured by trained health professionals or obtained through self-reports.

**Table 1:** Participating centers, study areas, and characteristics of the individual cohorts

Country and center	Individual EPIC cohort	Study area ESCAPE	Cohort size	Nature of cohort	No. of incident CD cases^a^	No. of incident UC cases^a^
Denmark, Aarhus, and Copenhagen	DCH	Copenhagen area	57,054	Population-based cohort of men and women aged 50–64 years. Enrollment period 1993–1997. Cases identified up to July 2007	11	30
France, regions throughout country	E3N	Paris, Lyon, Grenoble and, Marseille	72,996	Female teachers and co-workers aged 40–65 years from a nationwide health insurance scheme. Enrollment period 1993–1997. Cases identified up to April 2008	5	10
The Netherlands, Bilthoven	MORGEN	Large regions of the country	22,715	Population-based cohort of men and women aged 20–65 years. Enrollment period 1993–1997. Cases identified up to December 2009	9	27
The Netherlands, Utrecht	Prospect	Large regions of the country	17,357	Women aged 49–70 years in a breast cancer screening program. Enrollment period 1993–1997. Cases identified up to December 2009	7	14
The UK, Oxford	Oxford	Large regions of the country	57,498	Members of vegetarian societies and readers of health food magazines aged 20–80 years. Enrollment period 1993–2000. Cases identified up to May 2004	6	23
Total			227,620		38	104

*CD*, Crohn’s disease; *DCH*, Danish Diet, Cancer and Health cohort; *E3N*, Etude Épidémiologique des femmes de la Mutuelle Générale de l’Education Nationale; *EPIC*, European Prospective Investigation into Cancer and Nutrition; *ESCAPE,* European Study of Cohorts for Air Pollution Effects; *UC*, ulcerative colitis

^a^Only incident cases (and their controls) with air pollution estimates were selected

### Exposure Assessment

Air pollution data were obtained from the European Study of Cohorts for Air Pollution Effects (ESCAPE). The ESCAPE project was initiated to investigate the health effects of air pollution within European countries by linking exposure data to health data from ongoing cohort studies, including EPIC [17]. The study areas of ESCAPE generally included major cities with surrounding rural areas and included large regions of the country in some cohorts (Table 1). The methods of the standardized measurement procedures were published elsewhere [18, 19]. In summary, for each study area of ESCAPE, particulate matter with an aerodynamic diameter of <10 μm (PM_10_), particulate matter with an aerodynamic diameter of <2.5 μm (PM_2.5_), blackness of the PM_2.5_ exposed filter (PM_2.5 absorbance_) (a marker for black carbon or soot), nitrogen oxides (NO_*x*_) and NO_2_ were measured within 1 year during three different seasons between 2008 and 2011 in at least 20 sites [20, 21]. The concentration of PM_coarse_ was calculated as the difference between concentrations of PM_10_ and PM_2.5_. Subsequently, the annual average concentration of each pollutant was calculated. Air pollution exposure at the baseline residential address of study participants was then estimated by land-use regression models. These models were developed for each pollutant in each study area to explain the spatial variation of air pollution concentrations, taking various geographical factors into account, including altitude, population density, industrial land use, green space, and traffic flow variables. In addition to the air pollution concentrations, within ESCAPE two traffic indicators at the participant’s residential address were evaluated: traffic intensity on the nearest road (vehicles per day) and total traffic load on all major roads in a 100-m buffer (sum of traffic intensity multiplied by the length of those roads). Considering that air pollution was measured after enrollment, concentrations were back-extrapolated to the baseline year of each participant according to a standardized protocol used in previous ESCAPE work [17]. Back-extrapolation was only possible for pollutants for which historical data were available.

### Cases and Controls

The cohort was followed up until at least June 2004 and in some centers up to December 2009. Individuals with incident CD or UC were identified using population-based disease registries (Denmark, the Netherlands) or follow-up questionnaires (France, the UK). The medical reports of all potential cases were reviewed by local gastroenterologists to confirm the diagnoses and to obtain information on diagnostic investigations and disease extent. In this nested case–control analysis, each case was matched with four randomly selected unique controls for center, gender, age at recruitment (±6 months), and date of recruitment (±3 months). Controls were alive and had no CD, UC, microscopic or indeterminate colitis on the date of diagnosis of their matched case (incidence density sampling). For the present study, only cases with available air pollution estimates could be included. As the selection of controls was performed prior to the current study and the study areas of ESCAPE did not always cover the entire geographical area of the individual EPIC cohorts, air pollution measures were not available for all controls of a case.

### Statistical Analysis

Continuous data were expressed as medians with percentiles, because of the skewed distribution. Categorical data were expressed as frequencies with percentages. Correlations between the individual air pollution measures and traffic indicators were assessed using the Pearson correlation coefficient. Air pollutant concentrations were divided into tertiles according to the distribution across all matched controls. Conditional logistic regression analyses were performed to determine the association between air pollution exposure and IBD. Accordingly, associations were assessed within each cohort rather than between study areas. Considering the relatively small numbers of cases, associations were studied for CD and UC separately and combined. Odds ratios (ORs) with 95 % confidence intervals (CIs) were calculated to estimate the risk of IBD according to the different air pollutant concentrations and traffic indicators used as continuous and categorical variables. In a multivariable model, analyses were adjusted for smoking status (categorized into never smoker, former smoker, and current smoker) and socioeconomic status using educational level (categorized into primary school, technical school, secondary school, and higher education) as previous studies documented associations between these factors and IBD [22, 23]. All models for traffic indicators were additionally adjusted for background NO_2_ concentration. Trends across tertiles were computed using the median value of each tertile as a continuous variable. In sensitivity analyses, analyses were repeated for air pollutant concentrations back-extrapolated to the baseline year of participants. Two-sided *p* values below 0.05 were considered statistically significant. All analyses were performed using SPSS version 21 (IBM Corp., Armonk, NY, USA).

## Ethical Considerations

The original cohort studies were approved by the institutional medical ethics committees.

## Results

A total of 38 CD cases (median age at diagnosis 56.2 years, 76.3 % female) and 104 UC cases (median age at diagnosis 55.2 years, 70.2 % female) with air pollution measures were identified (Table 2). The median time between recruitment and diagnosis was 4.5 years [interquartile range (IQR) 2.3–6.8 years] and 3.8 years (IQR 2.1–6.4 years) for CD and UC, respectively. Fourteen CD cases (36.9 %) had ileal disease. Left-sided colitis (involvement limited up to the splenic flexure) was the commonest disease phenotype in UC cases (*n* = 45, 43.6 %). Air pollution estimates were available for 100, 90, 83, 70, and 27 % of controls from the Dutch Prospect cohort, the British cohort, the Dutch MORGEN cohort, the Danish cohort, and the French cohort, respectively. The distribution of air pollutant concentrations and traffic indicators for all IBD cases and controls per individual cohort is presented in Supplementary Tables 1–5. These tables show that within cohorts, pollution distributions were generally very similar between cases and controls and that in some cohorts, the concentration ranges of especially PM_2.5_ and PM_10_ were very small. The correlations between these estimates are presented in Supplementary Tables 6–10.

**Table 2:** Characteristics of inflammatory bowel disease cases and controls

	CD cases (*n* = 38)	Controls (*n* = 152)	UC cases (*n* = 104)	Controls (*n* = 416)
Female, *n* (%)	29 (76.3)	116 (76.3)	73 (70.2)	292 (70.2)
Age (years) at recruitment, median (IQR)	50.9 (44.7–60.9)	50.3 (44.5–59.0)	50.7 (43.2–56.1)	50.9 (43.2–56.6)
Age (years) at diagnosis, median (IQR)	56.2 (49.7–63.6)	–	55.2 (48.1–60.9)	–
Distribution of Crohn’s disease, *n* (%)				
L1, ileal	14 (36.9)	–	–	–
L2, colonic	13 (34.2)	–	–	–
L3, ileocolonic	10 (26.3)	–	–	–
+ L4, upper gastrointestinal disease	1 (2.6)	–	–	–
Unknown	0 (0.0)	–	–	–
Distribution of ulcerative colitis, *n* (%)				
E1, ulcerative proctitis	–	–	25 (24.3)	–
E2, left-sided colitis	–	–	45 (43.6)	–
E3, extensive colitis	–	–	26 (25.2)	–
Unknown	–	–	7 (6.8)	–
Smoking status at recruitment, *n* (%)				
Never smoker	13 (34.2)	52 (34.2)	36 (34.6)	180 (43.3)
Former smoker	10 (26.3)	54 (35.5)	34 (32.7)	118 (28.4)
Current smoker	15 (39.5)	44 (28.9)	33 (31.7)	116 (27.9)
Unknown	0 (0.0)	2 (1.3)	1 (1.0)	2 (0.5)
Highest educational level at recruitment, *n* (%)				
Primary school	4 (10.5)	29 (19.1)	15 (14.4)	61 (14.7)
Technical school	17 (44.7)	38 (25.0)	36 (34.6)	128 (30.8)
Secondary school	10 (26.3)	39 (25.7)	22 (21.2)	91 (21.9)
Higher education	7 (18.4)	45 (29.6)	27 (26.0)	116 (27.9)
Not specified	0 (0.0)	1 (0.7)	4 (3.8)	14 (3.4)
Unknown	0 (0.0)	0 (0.0)	0 (0.0)	6 (1.4)

*CD* Crohn’s disease, *IQR* interquartile range, *UC* ulcerative colitis

No statistically significant associations were detected between air pollution exposure and either CD (Table 3) or UC (Table 4), apart from a positive association for total traffic load on all major roads within a 100-m buffer [OR 1.58 (95 % CI 1.00–2.49) per 4,000,000 motor vehicles m per day] in the crude analysis of UC. The effect sizes were largely similar for the univariable and multivariable analyses and for CD and UC. In the crude analysis of CD and UC combined, residential exposure to PM_2.5_ was inversely associated with IBD, with an OR of 0.28 (95 % CI 0.08–0.94) per 5 μg/m^3^ (Table 5). A positive association was found for total traffic load on all major roads [OR 1.60 (95 % CI 1.06–2.43) per 4,000,000 motor vehicles × m per day]. These associations remained statistically significant with similar effect sizes after adjusting for smoking status and educational level in the multivariable analysis. Additionally, PM_10_ concentrations were inversely associated with IBD in the multivariable model, with an OR of 0.25 (95 % CI 0.08–0.78) per 10 μg/m^3^. Excluding data of the French cohort, which had the lowest proportion of IBD cases (*n* = 15) and controls with air pollution measures (*n* = 20), did not affect the direction of the effect sizes materially (data not shown). The associations for PM_2.5_ per cohort are presented in Supplementary Fig. 1. In the analysis based on tertiles, significant inverse associations were detected for PM_2.5_ (*p*_trend_ = 0.01) and PM_coarse_ (*p*_trend_ = 0.04) in IBD (Supplementary Table 11). Air pollution estimates back-extrapolated to the baseline year of each participant were generally higher than those during 2008–2011. Although no statistically significant associations were found, the sensitivity analyses for IBD for back-extrapolated concentrations of NO_2_, NO_*x*_, PM_2.5 absorbance_ and PM_10_ showed similar effect sizes as for nonback-extrapolated concentrations, apart from PM_2.5 absorbance_ (Table 5).

**Table 3:** Association between air pollution exposure and Crohn’s disease (*n* = 38 cases)

	Odds of CD	
	Unadjusted OR (95 % CI)	Adjusted OR (95 % CI)^a^
NO_2_	1.25 (0.68–2.30)	1.13 (0.59–2.16)
NO_*x*_	1.26 (0.75–2.12)	1.17 (0.67–2.03)
PM_2.5_	0.29 (0.03–2.71)	0.12 (0.01–1.77)
PM_2.5 absorbance_	0.59 (0.11–3.03)	0.34 (0.04–3.05)
PM_10_	0.45 (0.07–2.90)	0.10 (0.01–1.37)
PM_coarse_	1.07 (0.17–6.55)	0.59 (0.07–4.98)
Traffic intensity on the nearest road^b^	1.18 (0.82–1.70)	1.16 (0.79–1.71)
Traffic intensity on major roads within 100-m buffer^b^	1.92 (0.64–5.82)	1.81 (0.50–6.47)

ORs are presented for the following increments: 10 μg/m^3^ for NO_2_, 20 μg/m^3^ for NO_*x*_, 5 μg/m^3^ for PM_2.5_, 10^−5^ m^−1^ for PM_2.5 absorbance_, 10 μg/m^3^ for PM_10_, 5 μg/m^3^ for PM_coarse_, 5000 motor vehicles per day for the traffic intensity on the nearest road, 4,000,000 motor vehicles × m per day for the total traffic load on all major roads within a 100-m buffer

*CD* Crohn’s disease, *NO* nitrogen oxide, *OR* odds ratio, *PM* particulate matter

^a^Adjusted for smoking status and educational level

^b^Additionally adjusted for background NO_2_ concentration (continuous variable)

**Table 4:** Association between air pollution exposure and ulcerative colitis (*n* = 104 cases)

	Odds of UC	
	Unadjusted OR (95 % CI)	Adjusted OR (95 % CI)^a^
NO_2_	1.03 (0.73–1.46)	0.99 (0.70–1.41)
NO_*x*_	1.06 (0.79–1.43)	1.03 (0.76–1.38)
PM_2.5_	0.27 (0.06–1.17)	0.23 (0.05–1.02)
PM_2.5 absorbance_	1.19 (0.48–2.96)	1.20 (0.48–2.98)
PM_10_	0.34 (0.09–1.27)	0.28 (0.07–1.07)
PM_coarse_	0.38 (0.11–1.35)	0.31 (0.09–1.10)
Traffic intensity on the nearest road^b^	1.08 (0.90–1.29)	1.06 (0.88–1.27)
Traffic intensity on major roads within 100-m buffer^b^	1.58 (1.00–2.49)	1.55 (0.97–2.46)

ORs are presented for the following increments: 10 μg/m^3^ for NO_2_, 20 μg/m^3^ for NO_*x*_, 5 μg/m^3^ for PM_2.5_, 10^−5^ m^−1^ for PM_2.5 absorbance_, 10 μg/m^3^ for PM_10_, 5 μg/m^3^ for PM_coarse_, 5000 motor vehicles per day for the traffic intensity on the nearest road, 4,000,000 motor vehicles × m per day for the total traffic load on all major roads within a 100-m buffer

*NO* nitrogen oxide, *OR* odds ratio, *PM* particulate matter, *UC* ulcerative colitis

^a^Adjusted for smoking status and educational level

^b^Additionally adjusted for background NO_2_ concentration (continuous variable)

**Table 5:** Association between air pollution exposure and inflammatory bowel disease (*n* = 142 cases)

	Odds of IBD	
	Unadjusted OR (95 % CI)	Adjusted OR (95 % CI)^a^
NO_2_	1.08 (0.80–1.46)	1.05 (0.77–1.42)
NO_*x*_	1.11 (0.86–1.43)	1.08 (0.83–1.40)
PM_2.5_	0.28 (0.08–0.94)	0.24 (0.07–0.81)
PM_2.5 absorbance_	0.99 (0.45–2.17)	1.03 (0.45–2.34)
PM_10_	0.37 (0.13–1.09)	0.25 (0.08–0.78)
PM_coarse_	0.52 (0.19–1.45)	0.42 (0.14–1.20)
Traffic intensity on the nearest road^b^	1.09 (0.93–1.28)	1.07 (0.92–1.26)
Traffic intensity on major roads within 100-m buffer^b^	1.60 (1.06–2.43)	1.60 (1.04–2.46)
Back-extrapolated NO_2_	1.14 (0.78–1.66)	1.07 (0.73–1.59)
Back-extrapolated NO_*x*_	1.15 (0.86–1.55)	1.10 (0.81–1.49)
Back-extrapolated PM_2.5 absorbance_	0.46 (0.07–3.10)	0.40 (0.06–2.78)
Back-extrapolated PM_10_	0.20 (0.02–2.22)	0.17 (0.02–2.01)

ORs are presented for the following increments: 10 μg/m^3^ for NO_2_, 20 μg/m^3^ for NO_*x*_, 5 μg/m^3^ for PM_2.5_, 10^−5^ m^−1^ for PM_2.5 absorbance_, 10 μg/m^3^ for PM_10_, 5 μg/m^3^ for PM_coarse_, 5000 motor vehicles per day for the traffic intensity on the nearest road, 4,000,000 motor vehicles × m per day for the total traffic load on all major roads within a 100-m buffer

*IBD* inflammatory bowel disease, *NO* nitrogen oxide, *OR* odds ratio, *PM* particulate matter

^a^Adjusted for smoking status and educational level

^b^Additionally adjusted for background NO_2_ concentration (continuous variable)

## Discussion

In this European multicenter study, no consistent association between residential exposure to ambient air pollution and the risk of IBD was found. The effect sizes were mostly similar for CD and UC separately. Individuals with IBD were less likely to have higher exposure levels of PM_2.5_ and PM_10_, whereas a positive association was observed for total traffic load on all major nearby roads. Other air pollutants were mostly positively but not statistically significantly associated with IBD. Although not reaching statistical significance, the effect sizes were broadly similar when back-extrapolating air pollutant concentrations to the participants’ baseline year.

Numerous studies have confirmed the association of ambient air pollution with a variety of diseases, and several underlying biological mechanisms have been proposed [10, 17, 24, 25]. The link between air pollution and intestinal health is, however, less well established with a few studies showing positive associations with colorectal cancer, appendicitis, and nonspecific abdominal pain [26–30]. In this respect, the adverse effects of smoking [31, 32], which may have some resemblance with air pollution, may help indicate the potential impact of air pollution exposure. It is hypothesized that exposure to air pollutants may contribute to the development of intestinal disorders, including IBD, through diverse biological mechanisms. First, ingestion of air pollutants by inhalation or contaminated foods may have direct toxic effects on epithelial cells, which may induce proinflammatory responses and increase gut permeability [11]. Second, various studies have shown that air pollution exposure may result in systemic inflammatory effects, such as changes in circulating cytokines, including tumor necrosis factor alpha [33]. Notably, air pollution has also been related to immunologically mediated disorders that may share some epidemiological and pathogenic aspects with IBD [34]. For instance, exposure to PM_10_ has been associated with an increased risk of relapse in multiple sclerosis [35], whereas traffic exposure has been found to be positively associated with incident rheumatoid arthritis [36]. Third, it is thought that air pollution may modulate the microbial composition of the gut [14], as recently exemplified in two studies in which PM exposure in mice led to significant alterations in the microbiota and induced acute and chronic intestinal inflammatory responses [12, 13].

To date, very few epidemiological studies explored the effects of air pollution in IBD. In a nested case–control study from the UK, in the whole population, no significant association between air pollution and incident IBD was found [15]. However, children and young adults living in areas with higher levels (within the upper three quintiles) of NO_2_ and sulfur dioxide, respectively, were more likely to develop CD [OR 2.31 (95 % CI 1.25–4.28) for individuals aged ≤23 years] and UC [OR 2.00 (95 % CI 1.08–3.72) for individuals aged ≤25 years]. Land-use regression models for more refined exposure assessments could not be incorporated in this study. An ecological analysis from the US related hospitalizations for IBD to air pollution emissions from an emission inventory, but was unable to estimate air pollution concentrations [37]. In our study, which included mainly middle aged to elderly people, we observed opposing associations. This could indicate that different pollutants have differential effects in specific groups. For example, younger people (with a developing immune system) might be more susceptible to air pollutants as compared to older people. Importantly, children and young adults are also likely to spend more time outdoors and near their home, which may increase and better reflect true personal exposure. Exposure assessment may therefore be more accurate in these individuals. There were no statistically significant associations for CD and UC separately, which presumably resulted from the relatively small numbers of cases. Disease-specific results may exist, as previously observed, for example, in the opposing associations for smoking between CD and UC [22], although the effect sizes were mostly similar for both forms of IBD in our study. We observed inverse associations for some pollutants, which appeared largely consistent across centers and in the analyses using pollution exposure as continuous and categorical variables. It remains unclear whether these associations reflect a true causal relationship or that these pollutants are a proxy for other variables. Interestingly, in the earlier mentioned study, PM_10_ and NO_2_ concentrations were inversely associated with CD in individuals aged 44–57 years with ORs of 0.48 (95 % CI 0.29–0.80) and 0.56 (95 % CI 0.33–0.95), respectively [15], demonstrating that inverse associations have previously been observed in older patients in another population using a different methodological approach. Overall, despite previous data suggesting that air pollutants may negatively impact IBD, no coherent associations have currently been confirmed to draw firm conclusions on the putative adverse effects of air pollution exposure in CD and UC, indicating the need for further research.

This study had several strengths. First, the multicenter design enabled us to explore the association between air pollution and IBD in several European countries. Second, a significant advantage over previous studies was the use of land-use regression models to accurately determine small-scale variations in residential exposure to air pollution and traffic intensity for each participant according to a highly standardized protocol. Third, both cases and controls were drawn from the same baseline population, thereby reducing the risk of selection bias. Fourth, to ensure case ascertainment all diagnoses were confirmed by local physicians and participants with indeterminate or microscopic colitis were excluded. Fifth, the number of cases was similar to what was expected, which reduces the likelihood of follow-up bias [38].

Despite nesting in a well-characterized cohort, the limited sample size was an important drawback and our study may have been underpowered to detect statistical differences. Therefore, we chose to evaluate outcomes for CD and UC separately and for total IBD. Furthermore, air pollution estimates were assessed only at the residential address of each participant and not, for example, at their working address, where individuals may also be exposed. This could have resulted in misclassification of exposure. Moreover, air pollution was measured after diagnosis, which was a general issue in epidemiological studies using ESCAPE exposure data, such as in a previous study linking air pollution to mortality [17]. However, previous studies show that spatial air pollution contrasts remain stable over long periods of time and are well predicted with land-use regression models [39–43]. Using back-extrapolation, we evaluated the association for some pollutants at the baseline year of each participant, with effect sizes being broadly similar to the main analyses. It has been estimated that most of the participants did not move after enrollment [17], but exact data on moving were unavailable for all centers. The majority of cases developed IBD over the age of 50 years, indicating late-onset IBD, whereas CD and UC commonly present at an earlier age. This limited the opportunity to study age-specific effects, and the results may therefore not be generalizable to the whole IBD population as other phenotypes may have different environmental influences. Considering the limited sample size and multiple comparisons, we cannot entirely exclude that our results are due to chance findings. Finally, although we were able to either match or adjust for potential confounders, such as center and smoking, residual confounding (by unknown risk factors) could not be fully excluded in this observational study.

To conclude, we were unable to demonstrate a consistent association between residential exposure to ambient air pollution and IBD risk in this European multicenter cohort.

## Electronic supplementary material

Below is the link to the electronic supplementary material.

Supplementary Figure 1. Association between PM2.5 exposure and inflammatory bowel disease per cohort. CI: confidence interval; PM2.5: particulate matter with an aerodynamic diameter of less than 2.5 μm; OR: odds ratio. Adjustment for smoking status and educational level. ORs are presented for the following increment: 5 μg/m3 (TIFF 210 kb)

Supplementary material 2 (DOCX 97 kb)

## Abbreviations

CD: Crohn’s disease
CI: Confidence interval
EPIC: European Prospective Investigation into Cancer and Nutrition
ESCAPE: European Study of Cohorts for Air Pollution Effects
IBD: Inflammatory bowel disease
NO: Nitrogen oxide
OR: Odds ratio
PM: Particulate matter
UC: Ulcerative colitis
