# Supplementary figures and images for: Exposure to Ambient Air Pollution and the Risk of Inflammatory Bowel Disease: A European Nested Case–Control Study
Source: Dig Dis Sci. 2016 Jul 26;61(10):2963–71. doi: 10.1007/s10620-016-4249-4 (PMC5020109; doi:10.1007/s10620-016-4249-4)

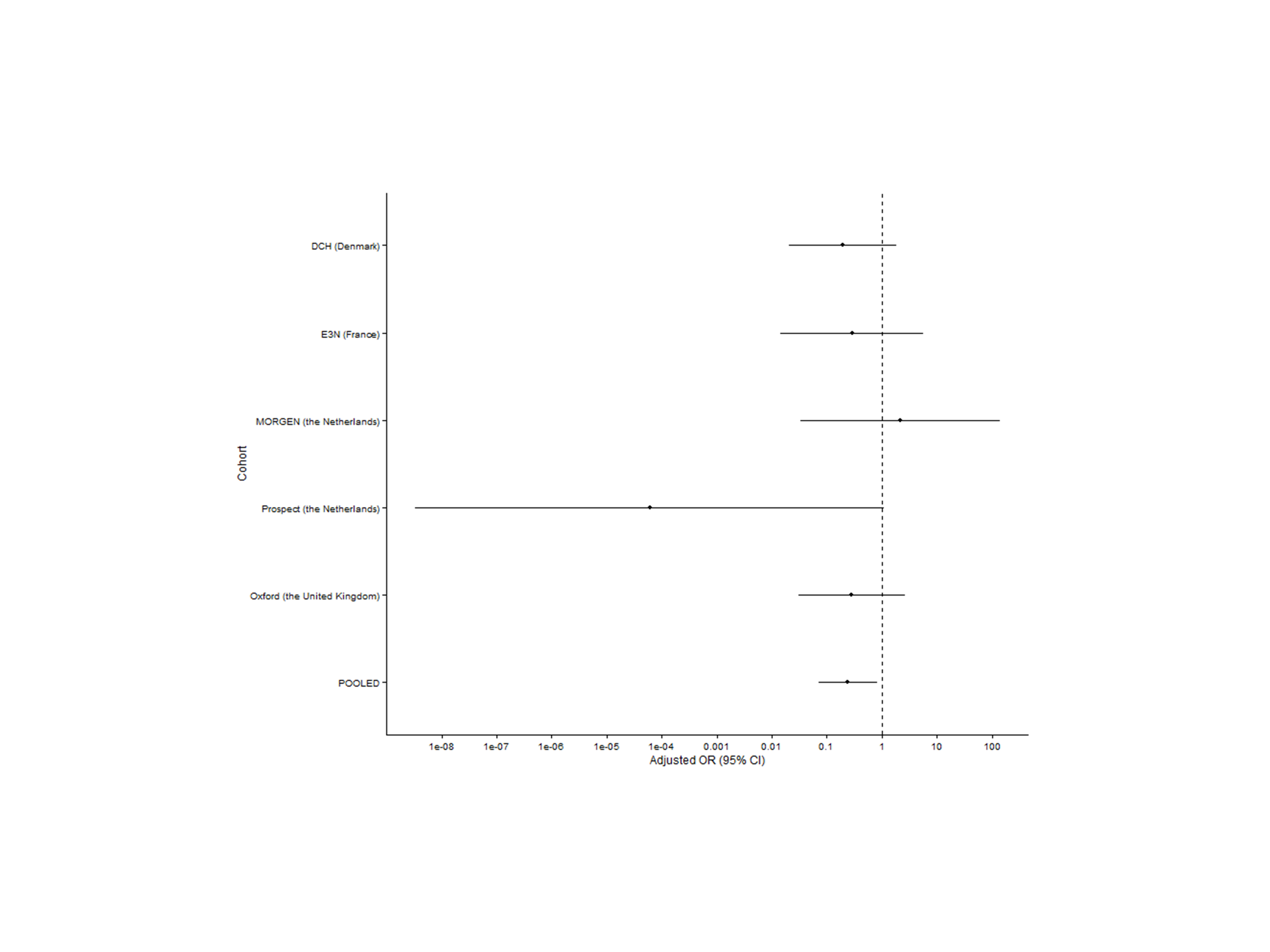

Supplement: Supplementary file 1 — Supplementary Figure 1. Association between PM2.5 exposure and inflammatory bowel disease per cohort. CI: confidence interval; PM2.5: particulate matter with an aerodynamic diameter of less than 2.5 μm; OR: odds ratio. Adjustment for smoking status and educational level. ORs are presented for the following increment: 5 μg/m3 (TIFF 210 kb) [file 10620_2016_4249_MOESM1_ESM.tif]
