# Supplementary material for: Exposure to Ambient Air Pollution and the Risk of Inflammatory Bowel Disease: A European Nested Case–Control Study
Source: Dig Dis Sci. 2016 Jul 26;61(10):2963–71. doi: 10.1007/s10620-016-4249-4 (PMC5020109; doi:10.1007/s10620-016-4249-4)

**Supplementary Table 1.** Air pollutant concentrations of inflammatory bowel disease cases and controls in the DCH cohort (Denmark)

|  | **n** | **Min** | **5^th^ percentile** | **10^th^ percentile** | **Median** | **90^th^ percentile** | **95^th^ percentile** | **Max** |
| --- | --- | --- | --- | --- | --- | --- | --- | --- |
| **IBD cases**  NO_2_ (μg/m^3^)  NO_x_ (μg/m^3^)  PM_2.5_ (μg/m^3^)  PM_2.5 absorbance_ (10^–5^/m)  PM_10_ (μg/m^3^)  PM_coarse_ (μg/m^3^)  Traffic intensity on the nearest road*  Traffic intensity on major roads within 100 m buffer** | 41  41  41  41  41  41  41  41 | 5.72  1.78  7.98  0.70  12.6  3.97  200  0.00 | 6.04  2.43  8.30  0.71  12.7  4.09  200  0.00 | 8.64  8.94  9.49  0.81  13.9  4.29  200  0.00 | 15.5  22.9  11.4  1.23  16.9  5.84  245  0.00 | 27.8  62.0  12.4  1.52  20.1  7.11  14,935  5,691,849 | 38.3  83.5  12.8  1.63  21.1  7.33  28,830  14,933,343 | 42.1  91.2  12.8  1.70  21.3  7.37  81,300  16,271,745 |
| **Controls**  NO_2_ (μg/m^3^)  NO_x_ (μg/m^3^)  PM_2.5_ (μg/m^3^)  PM_2.5 absorbance_ (10^–5^/m)  PM_10_ (μg/m^3^)  PM_coarse_ (μg/m^3^)  Traffic intensity on the nearest road*  Traffic intensity on major roads within 100 m buffer** | 115  115  115  115  115  115  115  115 | 6.11  2.53  8.35  0.66  12.6  3.97  200  0.00 | 8.16  7.03  9.50  0.81  13.3  4.00  200  0.00 | 8.79  9.23  10.4  0.84  14.5  4.32  200  0.00 | 15.1  21.7  11.5  1.13  17.0  5.75  245  0.00 | 28.0  62.2  12.4  1.41  19.6  7.42  9,179  3,495,665 | 29.5  64.1  12.7  1.49  20.5  7.73  17,310  4,802,733 | 35.7  78.2  12.8  1.65  22.3  8.43  39,600  11,714,652 |

DCH: Danish Diet, Cancer and Health; NO: nitrogen oxide: PM: particulate matter. *Motor vehicles per day. ** Vehicles*m per day

**Supplementary Table 2.** Air pollutant concentrations of inflammatory bowel disease cases and controls in the E3N cohort (France)

|  | **n** | **Min** | **10^th^ percentile** | **Median** | **90^th^ percentile** | **Max** |
| --- | --- | --- | --- | --- | --- | --- |
| **IBD cases**  NO_2_ (μg/m^3^)  NO_x_ (μg/m^3^)  PM_2.5_ (μg/m^3^)  PM_2.5 absorbance_ (10^–5^/m)  PM_10_ (μg/m^3^)  PM_coarse_ (μg/m^3^)  Traffic intensity on the nearest road*  Traffic intensity on major roads within 100 m buffer** | 15  15  14  13  14  14  15  15 | 13.5  22.7  12.0  0.94  19.6  4.93  79.0  0.00 | 14.3  24.2  12.1  0.95  19.6  5.21  404  0.00 | 23.8  34.5  14.1  1.50  23.8  7.00  3,685  0.00 | 56.8  109  16.7  3.03  32.5  12.9  10,535  4,529,232 | 64.3  127  17.6  3.45  35.2  13.7  11,371  6,387,672 |
| **Controls**  NO_2_ (μg/m^3^)  NO_x_ (μg/m^3^)  PM_2.5_ (μg/m^3^)  PM_2.5 absorbance_ (10^–5^/m)  PM_10_ (μg/m^3^)  PM_coarse_ (μg/m^3^)  Traffic intensity on the nearest road*  Traffic intensity on major roads within 100 m buffer** | 20  20  17  16  17  17  20  20 | 13.5  25.6  12.4  0.94  19.6  4.58  793  0.00 | 14.4  31.8  12.9  1.02  20.1  4.59  878  0.00 | 23.5  38.1  14.7  1.64  23.4  7.72  6,835  95,186 | 38.9  88.1  17.6  3.05  32.8  11.8  14,667  5,045,816 | 48.2  100  19.5  3.09  39.1  11.8  38,903  6,463,042 |

E3N: E3N: Etude Épidémiologique des femmes de la Mutuelle Générale de l’Education Nationale; NO: nitrogen oxide: PM: particulate matter. *Motor vehicles per day. ** Vehicles*m per day

**Supplementary Table 3.** Air pollutant concentrations of inflammatory bowel disease cases and controls in the MORGEN cohort (the Netherlands)

|  | **n** | **Min** | **5^th^ percentile** | **10^th^ percentile** | **Median** | **90^th^ percentile** | **95^th^ percentile** | **Max** |
| --- | --- | --- | --- | --- | --- | --- | --- | --- |
| **IBD cases**  NO_2_ (μg/m^3^)  NO_x_ (μg/m^3^)  PM_2.5_ (μg/m^3^)  PM_2.5 absorbance_ (10^–5^/m)  PM_10_ (μg/m^3^)  PM_coarse_ (μg/m^3^)  Traffic intensity on the nearest road*  Traffic intensity on major roads within 100 m buffer** | 36  36  36  36  36  36  36  36 | 15.7  24.2  16.1  1.08  23.8  7.69  0.00  0.00 | 16.3  24.7  16.3  1.10  23.9  7.70  0.00  0.00 | 17.0  25.7  16.4  1.10  24.0  7.71  50.0  0.00 | 20.3  30.5  16.8  1.34  24.6  8.00  230  0.00 | 36.0  56.2  17.4  1.61  27.7  9.81  5,672  4,355,093 | 40.2  78.9  17.9  1.82  28.7  10.6  16,714  7,176,805 | 46.0  86.0  20.1  2.49  28.9  10.8  21,111  10,354,235 |
| **Controls**  NO_2_ (μg/m^3^)  NO_x_ (μg/m^3^)  PM_2.5_ (μg/m^3^)  PM_2.5 absorbance_ (10^–5^/m)  PM_10_ (μg/m^3^)  PM_coarse_ (μg/m^3^)  Traffic intensity on the nearest road*  Traffic intensity on major roads within 100 m buffer** | 120  120  120  120  120  120  120  120 | 13.8  21.7  16.0  1.03  23.8  7.61  0.00  0.00 | 16.1  24.4  16.3  1.10  24.0  7.71  25.1  0.00 | 17.2  25.2  16.4  1.11  24.0  7.76  70.2  0.00 | 20.5  31.2  16.7  1.33  24.6  8.11  372  0.00 | 29.9  48.0  17.4  1.52  26.6  9.83  2,467  1,682,024 | 35.4  57.4  17.5  1.64  28.5  10.8  5,444  3,376,439 | 41.7  77.6  19.3  2.23  31.4  12.0  34,363  8,312,163 |

NO: nitrogen oxide: PM: particulate matter. *Motor vehicles per day. ** Vehicles*m per day

**Supplementary Table 4.** Air pollutant concentrations of inflammatory bowel disease cases and controls in the Prospect cohort (the Netherlands)

|  | **n** | **Min** | **5^th^ percentile** | **10^th^ percentile** | **Median** | **90^th^ percentile** | **95^th^ percentile** | **Max** |
| --- | --- | --- | --- | --- | --- | --- | --- | --- |
| **IBD cases**  NO_2_ (μg/m^3^)  NO_x_ (μg/m^3^)  PM_2.5_ (μg/m^3^)  PM_2.5 absorbance_ (10^–5^/m)  PM_10_ (μg/m^3^)  PM_coarse_ (μg/m^3^)  Traffic intensity on the nearest road*  Traffic intensity on major roads within 100 m buffer** | 21  21  21  21  21  21  21  21 | 17.4  25.8  16.2  1.08  23.9  7.67  0.00  0.00 | 17.6  26.2  16.2  1.08  23.9  7.67  2.40  0.00 | 20.3  30.3  16.3  1.12  24.0  7.69  25.6  0.00 | 24.7  35.1  16.6  1.26  24.8  8.16  244  0.00 | 32.9  48.4  17.1  1.63  27.1  9.09  483  4,066,857 | 34.5  68.9  17.4  1.66  27.3  9.35  568  6,087,546 | 34.6  71.2  17.4  1.66  27.3  9.38  576  6,286,026 |
| **Controls**  NO_2_ (μg/m^3^)  NO_x_ (μg/m^3^)  PM_2.5_ (μg/m^3^)  PM_2.5 absorbance_ (10^–5^/m)  PM_10_ (μg/m^3^)  PM_coarse_ (μg/m^3^)  Traffic intensity on the nearest road*  Traffic intensity on major roads within 100 m buffer** | 84  84  84  84  84  84  84  84 | 18.9  25.6  16.2  1.13  23.9  7.60  0.00  0.00 | 20.1  29.4  16.3  1.17  24.1  7.69  0.00  0.00 | 20.8  30.3  16.4  1.20  24.3  7.80  32.2  0.00 | 27.7  37.7  16.7  1.35  25.3  8.33  305  0.00 | 33.0  59.0  17.6  1.63  27.2  9.60  1,464  1,917,725 | 33.6  70.3  18.0  1.78  27.8  9.99  3,488  3,199,929 | 38.1  79.1  19.3  2.02  29.2  12.2  33,423  6,565,291 |

NO: nitrogen oxide: PM: particulate matter. *Motor vehicles per day. ** Vehicles*m per day

**Supplementary Table 5.** Air pollutant concentrations of inflammatory bowel disease cases and controls in the Oxford cohort (the United Kingdom)

|  | **n** | **Min** | **5^th^ percentile** | **10^th^ percentile** | **Median** | **90^th^ percentile** | **95^th^ percentile** | **Max** |
| --- | --- | --- | --- | --- | --- | --- | --- | --- |
| **IBD cases**  NO_2_ (μg/m^3^)  NO_x_ (μg/m^3^)  PM_2.5_ (μg/m^3^)  PM_2.5 absorbance_ (10^–5^/m)  PM_10_ (μg/m^3^)  PM_coarse_ (μg/m^3^)  Traffic intensity on the nearest road*  Traffic intensity on major roads within 100 m buffer** | 29  29  29  29  29  29  29  29 | 13.1  20.0  8.19  0.84  12.7  5.65  500  0.00 | 13.2  20.5  8.23  0.84  13.3  5.67  500  0.00 | 14.7  21.2  8.45  0.85  14.1  5.70  500  0.00 | 24.0  39.8  9.72  1.06  15.9  5.96  500  0.00 | 35.7  60.2  11.0  1.36  17.7  6.92  500  0.00 | 37.1  64.3  11.0  1.91  19.0  8.06  9,021  863,972 | 37.1  66.0  11.0  1.93  20.1  9.11  9,321  1,727,944 |
| **Controls**  NO_2_ (μg/m^3^)  NO_x_ (μg/m^3^)  PM_2.5_ (μg/m^3^)  PM_2.5 absorbance_ (10^–5^/m)  PM_10_ (μg/m^3^)  PM_coarse_ (μg/m^3^)  Traffic intensity on the nearest road*  Traffic intensity on major roads within 100 m buffer** | 104  104  104  104  104  104  104  104 | 12.9  19.8  8.17  0.83  12.2  5.59  500  0.00 | 13.2  21.2  8.26  0.85  13.1  5.67  500  0.00 | 15.3  25.0  8.56  0.86  14.4  5.72  500  0.00 | 23.2  40.1  9.89  1.02  16.2  6.14  500  0.00 | 36.4  63.7  11.3  1.46  20.4  9.05  500  1,760,577 | 41.2  80.7  12.4  1.90  21.3  9.10  10,284  2,939,042 | 53.1  116  13.5  2.27  22.7  9.18  18,281  4,575,984 |

NO: nitrogen oxide: PM: particulate matter. *Motor vehicles per day. ** Vehicles*m per day

**Supplementary Table 6.** Correlation matrix for all individual measures in the DCH cohort (Denmark)

|  | Traffic intensity on the nearest road | Traffic intensity on major roads within 100 m buffer | NO_2_ | NO_X_ | PM_2.5_ | PM_2.5 absorbance_ | PM_10_ | PM_coarse_ | Back-extra-polated NO_2_ | Back-extra-polated NO_X_ |
| --- | --- | --- | --- | --- | --- | --- | --- | --- | --- | --- |
| Traffic intensity on the nearest road | 1  156 | *🡨 r*  *🡨 N* |  |  |  |  |  |  |  |  |
| Traffic intensity on major roads within 100 m buffer | 0.72  156 | 1  156 |  |  |  |  |  |  |  |  |
| NO_2_ | 0.64  156 | 0.70  156 | 1  156 |  |  |  |  |  |  |  |
| NO_X_ | 0.71  156 | 0.67  156 | 0.97  156 | 1  156 |  |  |  |  |  |  |
| PM_2.5_ | 0.20  156 | 0.30  156 | 0.56  156 | 0.48  156 | 1  156 |  |  |  |  |  |
| PM_2.5 absorbance_ | 0.43  156 | 0.53  156 | 0.68  156 | 0.63  156 | 0.48  156 | 1  156 |  |  |  |  |
| PM_10_ | 0.33  156 | 0.47  156 | 0.77  156 | 0.67  156 | 0.76  156 | 0.70  156 | 1  156 |  |  |  |
| PM_coarse_ | 0.42  156 | 0.43  156 | 0.71  156 | 0.69  156 | 0.57  156 | 0.57  156 | 0.63  156 | 1  156 |  |  |
| Back-extrapolated NO_2_ | 0.65  156 | 0.70  156 | 0.99  156 | 0.97  156 | 0.53  156 | 0.68  156 | 0.75  156 | 0.66  156 | 1  156 |  |
| Back-extrapolated NO_X_ | 0.71  156 | 0.68  156 | 0.96  156 | 1.00  156 | 0.46  156 | 0.62  156 | 0.66  156 | 0.66  156 | 0.97  156 | 1  156 |

DCH: Danish Diet, Cancer and Health; NO: nitrogen oxide; PM: particulate matter.

Correlations assessed using Pearson correlation coefficient *r*. *N* indicates numbers of subjects.

**Supplementary Table 7.** Correlation matrix for all individual measures in the E3N cohort (France)

|  | Traffic intensity on the nearest road | Traffic intensity on major roads within 100 m buffer | NO_2_ | NO_X_ | PM_2.5_ | PM_2.5 absorbance_ | PM_10_ | PM_coarse_ |
| --- | --- | --- | --- | --- | --- | --- | --- | --- |
| Traffic intensity on the nearest road | 1  35 | *🡨 r*  *🡨 N* |  |  |  |  |  |  |
| Traffic intensity on major roads within 100 m buffer | 0.44  35 | 1  35 |  |  |  |  |  |  |
| NO_2_ | 0.38  35 | 0.70  35 | 1  35 |  |  |  |  |  |
| NO_X_ | 0.46  35 | 0.72  35 | 0.91  35 | 1  35 |  |  |  |  |
| PM_2.5_ | 0.67  31 | 0.40  31 | 0.63  31 | 0.53  31 | 1  31 |  |  |  |
| PM_2.5 absorbance_ | 0.28  29 | 0.81  29 | 0.80  29 | 0.62  29 | 0.57  29 | 1  29 |  |  |
| PM_10_ | 0.65  31 | 0.33  31 | 0.57  31 | 0.56  31 | 0.78  31 | 0.25  29 | 1  31 |  |
| PM_coarse_ | 0.25  31 | 0.60  31 | 0.89  31 | 0.73  31 | 0.65  31 | 0.76  29 | 0.61  31 | 1  31 |

E3N: E3N: Etude Épidémiologique des femmes de la Mutuelle Générale de l’Education Nationale; NO: nitrogen oxide; PM: particulate matter.

Correlations assessed using Pearson correlation coefficient *r*. *N* indicates numbers of subjects.

**Supplementary Table 8.** Correlation matrix for all individual measures in the MORGEN cohort (the Netherlands)

|  | Traffic intensity on the nearest road | Traffic intensity on major roads within 100 m buffer | NO_2_ | NO_X_ | PM_2.5_ | PM_2.5 absorbance_ | PM_10_ | PM_coarse_ | Back-extra-polated NO_2_ | Back-extra-polated NO_X_ | Back-extra-polated PM_2.5 absorbance_ | Back-extra-polated PM_10_ |
| --- | --- | --- | --- | --- | --- | --- | --- | --- | --- | --- | --- | --- |
| Traffic intensity on the nearest road | 1  156 | *🡨 r*  *🡨 N* |  |  |  |  |  |  |  |  |  |  |
| Traffic intensity on major roads within 100 m buffer | 0.59  156 | 1  156 |  |  |  |  |  |  |  |  |  |  |
| NO_2_ | 0.51  156 | 0.67  156 | 1  156 |  |  |  |  |  |  |  |  |  |
| NO_X_ | 0.59  156 | 0.62  156 | 0.88  156 | 1  156 |  |  |  |  |  |  |  |  |
| PM_2.5_ | 0.46  156 | 0.38  156 | 0.31  156 | 0.47  156 | 1  156 |  |  |  |  |  |  |  |
| PM_2.5 absorbance_ | 0.54  156 | 0.62  156 | 0.77  156 | 0.83  156 | 0.81  156 | 1  156 |  |  |  |  |  |  |
| PM_10_ | 0.48  156 | 0.64  156 | 0.91  156 | 0.85  156 | 0.44  156 | 0.84  156 | 1  156 |  |  |  |  |  |
| PM_coarse_ | 0.48  156 | 0.61  156 | 0.91  156 | 0.82  156 | 0.24  156 | 0.70  156 | 0.93  156 | 1  156 |  |  |  |  |
| Back-extrapolated NO_2_ | 0.52  156 | 0.67  156 | 0.99  156 | 0.88  156 | 0.32  156 | 0.78  156 | 0.91  156 | 0.90  156 | 1  156 |  |  |  |
| Back-extrapolated NO_X_ | 0.60  156 | 0.63  156 | 0.87  156 | 0.99  156 | 0.48  156 | 0.83  156 | 0.85  156 | 0.81  156 | 0.88  156 | 1  156 |  |  |
| Back-extrapolated PM_2.5 absorbance_ | 0.51  156 | 0.55  156 | 0.65  156 | 0.71  156 | 0.77  156 | 0.90  156 | 0.72  156 | 0.56  156 | 0.67  156 | 0.76  156 | 1  156 |  |
| Back-extrapolated PM_10_ | 0.36  156 | 0.45  156 | 0.54  156 | 0.54  156 | 0.42  156 | 0.59  156 | 0.61  156 | 0.50  156 | 0.59  156 | 0.63  156 | 0.83  156 | 1  156 |

NO: nitrogen oxide; PM: particulate matter.

Correlations assessed using Pearson correlation coefficient *r*. *N* indicates numbers of subjects.

**Supplementary Table 9.** Correlation matrix for all individual measures in the Prospect cohort (the Netherlands)

|  | Traffic intensity on the nearest road | Traffic intensity on major roads within 100 m buffer | NO_2_ | NO_X_ | PM_2.5_ | PM_2.5 absorbance_ | PM_10_ | PM_coarse_ | Back-extra-polated NO_2_ | Back-extra-polated NO_X_ | Back-extra-polated PM_2.5 absorbance_ | Back-extra-polated PM_10_ |
| --- | --- | --- | --- | --- | --- | --- | --- | --- | --- | --- | --- | --- |
| Traffic intensity on the nearest road | 1  105 | *🡨 r*  *🡨 N* |  |  |  |  |  |  |  |  |  |  |
| Traffic intensity on major roads within 100 m buffer | 0.42  105 | 1  105 |  |  |  |  |  |  |  |  |  |  |
| NO_2_ | 0.26  105 | 0.43  105 | 1  105 |  |  |  |  |  |  |  |  |  |
| NO_X_ | 0.16  105 | 0.32  105 | 0.77  105 | 1  105 |  |  |  |  |  |  |  |  |
| PM_2.5_ | 0.28  105 | 0.43  105 | 0.50  105 | 0.60  105 | 1  105 |  |  |  |  |  |  |  |
| PM_2.5 absorbance_ | 0.20  105 | 0.46  105 | 0.80  105 | 0.81  105 | 0.84  105 | 1  105 |  |  |  |  |  |  |
| PM_10_ | 0.14  105 | 0.44  105 | 0.81  105 | 0.86  105 | 0.77  105 | 0.97  105 | 1  105 |  |  |  |  |  |
| PM_coarse_ | 0.46  105 | 0.46  105 | 0.69  105 | 0.71  105 | 0.63  105 | 0.64  105 | 0.68  105 | 1  105 |  |  |  |  |
| Back-extrapolated NO_2_ | 0.24  105 | 0.41  105 | 0.99  105 | 0.77  105 | 0.50  105 | 0.79  105 | 0.81  105 | 0.69  105 | 1  105 |  |  |  |
| Back-extrapolated NO_X_ | 0.15  105 | 0.30  105 | 0.75  105 | 0.99  105 | 0.59  105 | 0.79  105 | 0.85  105 | 0.69  105 | 0.77  105 | 1  105 |  |  |
| Back-extrapolated PM_2.5 absorbance_ | 0.12  105 | 0.36  105 | 0.70  105 | 0.68  105 | 0.72  105 | 0.88  105 | 0.85  105 | 0.50  105 | 0.74  105 | 0.72  105 | 1  105 |  |
| Back-extrapolated PM_10_ | -0.07  105 | 0.12  105 | 0.40  105 | 0.41  105 | 0.37  105 | 0.50  105 | 0.52  105 | 0.23  105 | 0.50  105 | 0.50  105 | 0.81  105 | 1  105 |

NO: nitrogen oxide; PM: particulate matter.

Correlations assessed using Pearson correlation coefficient *r*. *N* indicates numbers of subjects.

**Supplementary Table 10.** Correlation matrix for all individual measures in the Oxford cohort (the United Kingdom)

|  | Traffic intensity on the nearest road | Traffic intensity on major roads within 100 m buffer | NO_2_ | NO_X_ | PM_2.5_ | PM_2.5 absorbance_ | PM_10_ | PM_coarse_ | Back-extra-polated NO_2_ | Back-extra-polated NO_X_ |
| --- | --- | --- | --- | --- | --- | --- | --- | --- | --- | --- |
| Traffic intensity on the nearest road | 1  133 | *🡨 r*  *🡨 N* |  |  |  |  |  |  |  |  |
| Traffic intensity on major roads within 100 m buffer | 0.68  133 | 1  133 |  |  |  |  |  |  |  |  |
| NO_2_ | 0.41  133 | 0.51  133 | 1  133 |  |  |  |  |  |  |  |
| NO_X_ | 0.47  133 | 0.58  133 | 0.95  133 | 1  133 |  |  |  |  |  |  |
| PM_2.5_ | 0.33  133 | 0.45  133 | 0.92  133 | 0.91  133 | 1  133 |  |  |  |  |  |
| PM_2.5 absorbance_ | 0.47  133 | 0.54  133 | 0.79  133 | 0.71  133 | 0.65  133 | 1  133 |  |  |  |  |
| PM_10_ | 0.36  133 | 0.36  133 | 0.50  133 | 0.47  133 | 0.52  133 | 0.61  133 | 1  133 |  |  |  |
| PM_coarse_ | 0.28  133 | 0.28  133 | 0.22  133 | 0.19  133 | 0.17  133 | 0.52  133 | 0.83  133 | 1  133 |  |  |
| Back-extrapolated NO_2_ | 0.50  51 | 0.58  51 | 1.00  51 | 0.93  51 | 0.92  51 | 0.86  51 | 0.54  51 | 0.31  51 | 1  51 |  |
| Back-extrapolated NO_X_ | 0.55  51 | 0.65  51 | 0.93  51 | 0.99  51 | 0.92  51 | 0.80  51 | 0.54  51 | 0.30  51 | 0.94  51 | 1  51 |

NO: nitrogen oxide; PM: particulate matter.

Correlations assessed using Pearson correlation coefficient *r*. *N* indicates numbers of subjects.

**Supplementary Table 11.** Odds of inflammatory bowel disease according to tertile of air pollutant exposure

| Air pollutant | Tertile (ranges) | IBD cases | Controls | Unadjusted OR (95% CI) | Adjusted OR (95% CI)* |
| --- | --- | --- | --- | --- | --- |
| NO_2_ (μg/m^3^) | 1 (5.72-19.0)  2 (19.1-25.6)  3 (25.7-64.3) | 51  48  43 | 148  148  147 | 1.00  1.02 (0.58-1.79)  0.95 (0.52-1.74)  p _trend_ = 0.85 | 1.00  1.03 (0.58-1.82)  0.91 (0.50-1.65)  p _trend_ = 0.71 |
| NO_x_ (μg/m^3^) | 1 (1.78-28.5)  2 (28.6-39.3)  3 (39.4-127) | 49  41  52 | 148  147  148 | 1.00  0.84 (0.47-1.52)  1.16 (0.66-2.06)  p _trend_ = 0.44 | 1.00  0.80 (0.43-1.47)  1.02 (0.56-1.84)  p _trend_ = 0.74 |
| PM_2.5_ (μg/m^3^) | 1 (7.98-11.4)  2 (11.5-16.6)  3 (16.7-20.1) | 48  60  33 | 146  147  147 | 1.00  0.69 (0.33-1.46)  0.24 (0.08-0.71)  p _trend_ = 0.01 | 1.00  0.71 (0.33-1.50)  0.26 (0.08-0.78)  p _trend_ = 0.01 |
| PM_2.5 absorbance_  (10^-5^m^-1^) | 1 (0.66-1.13)  2 (1.14-1.35)  3 (1.36-3.45) | 47  50  43 | 146  147  146 | 1.00  1.10 (0.66-1.86)  0.76 (0.41-1.42)  p _trend_ = 0.39 | 1.00  1.14 (0.67-1.96)  0.72 (0.38-1.37)  p _trend_ = 0.32 |
| PM_10_ (μg/m^3^) | 1 (12.2-17.5)  2 (17.6-24.5)  3 (24.6-39.1) | 50  49  42 | 147  146  147 | 1.00  0.73 (0.37-1.44)  0.58 (0.22-1.52)  p _trend_ = 0.26 | 1.00  0.71 (0.36-1.41)  0.46 (0.17-1.26)  p _trend_ = 0.15 |
| PM_coarse_ (μg/m^3^) | 1 (3.97-6.32)  2 (6.33-8.08)  3 (8.09-13.7) | 56  50  35 | 146  146  148 | 1.00  0.86 (0.46-1.61)  0.48 (0.22-1.01)  p _trend_ = 0.09 | 1.00  0.81 (0.43-1.54)  0.40 (0.18-0.87)  p _trend_ = 0.04 |

IBD: inflammatory bowel disease; NO: nitrogen oxide; OR: odds ratio; PM: particulate matter.

*adjusted for smoking status and educational level


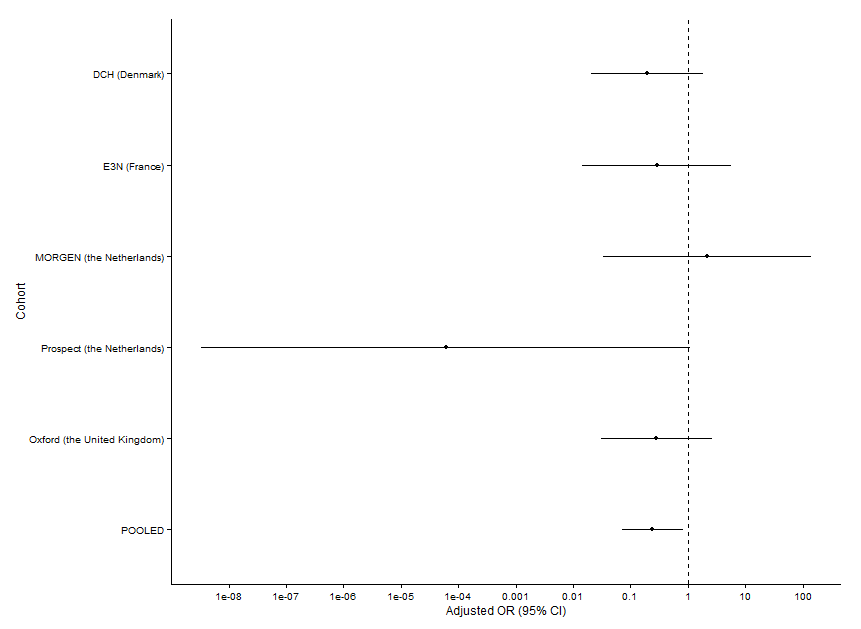

Supplement: Supplementary file 2 — Supplementary material 2 (DOCX 97 kb) [file 10620_2016_4249_MOESM2_ESM.docx]
